# Does the Environment Influence the Frequency of Concussion Incidence in Professional Football?

**DOI:** 10.7759/cureus.3627

**Published:** 2018-11-23

**Authors:** Syed Haider, Halley P Kaye-Kauderer, Akbar Y Maniya, Jennifer B Dai, Adam Y Li, Alexander F Post, Stanislaw Sobotka, Ryan Adams, Alex Gometz, Mark R Lovell, Tanvir F Choudhri

**Affiliations:** 1 Neurosurgery, The Icahn School of Medicine at Mount Sinai, New York, USA; 2 Surgery, Montefiore Medical Center, New York, USA; 3 Sports Medicine, Concussion Management of New York, New York, USA; 4 Neurology, University of Pittsburgh Medical Center, Pittsburgh , USA

**Keywords:** concussion, epidemiology, american football, temperature, environmental factors

## Abstract

Background

Sports-related concussion is a major cause of mild traumatic brain injury (mTBI). It is possible that environmental factors, such as temperature, humidity, and stadium's altitude, may influence the overall incidence of concussions during a game.

Purpose

To examine the impact of environmental factors, such as temperature, humidity, barometric pressure, and dew point, on concussion incidence.

Methods

Public Broadcasting Service (PBS) FRONTLINE Concussion Watch was used to collect injury data on 32 NFL teams during regular season games from 2012 to 2015. Weather data points were collected from Weather Underground. Concussion incidence per game, the probability of a concussion during a game, and a difference in mean game-day temperature, humidity, dew point, and barometric pressure between concussion and concussion-free games were calculated. Our analysis included t-tests, analysis of variance (ANOVA), multivariate correlation tests, and logistic and Poisson regression.

Results

Overall, 564 concussions were reported. There were 411 games with concussions and 549 games without concussions. We observed a significant decrease in concussion incidence with increasing temperature, both when the temperature was divided into 20^o^F increments or into quartiles (p = 0.005 and p = 0.002, respectively). We identified a statistically significant lower mean-game day temperature in concussion games compared to concussion-free games (p < 0.0006). We also observed a significant decrease in the incidence of concussion per game with increasing dew point. There was no significant difference in concussion incidence in barometric pressure and humidity. The logistic regression model predicted a decrease in the probability of a concussion in games with higher temperatures and dew points.

Conclusions

National Football League (NFL) players experienced an increased risk of concussion during football games played in colder temperatures and at lower dew points. Further research on environmental effects on concussions may aid in improving player safety in football leagues.

## Introduction

In the last decade, there has been significant attention to sports-related concussions as a major cause of mild traumatic brain injury (mTBI) [1]. Media outlets, professional sports organizations, physicians, families, and athletes have raised concerns about the causes and implications of concussions in professional and amateur sports leagues. This has led to equipment improvements, resulting in better concussion reporting protocols in high-profile leagues [2], such as the National Football League (NFL) and National Basketball Association (NBA). Additionally, educational campaigns have been designed to reduce concussion incidence and games missed due to concussion [3].

Previous studies have found that environmental factors play a role in recovery from mild traumatic brain injury (mTBI) [4-7]. However, it is less clear whether environmental factors play a role in the initial incidence of concussions. Some recent studies have shown that the environmental conditions of altitude and temperature may or may not affect concussion incidence in American football [8-11]. Other environmental factors have not been studied to the best of our knowledge. Examining additional environmental and physiological factors may help researchers further evaluate an individual’s risk of concussion and, by doing so, it can also help to develop new strategies or therapies to lower their incidence across sports leagues.

Our study seeks to explore the role that temperature, humidity, dew point, and barometric pressure play in the prevalence of concussions in the NFL over four seasons from 2012 to 2015. Previous studies on NFL concussions included limited environmental and extrinsic variables [8], so the goal of this study is to increase the understanding of the impact of environmental conditions on concussion risk.

## Materials and methods

The official injury report data was collected from Public Broadcasting Service (PBS) FRONTLINE Concussion Watch, a publically available website (http://www.pbs.org/wgbh/pages/frontline/concussion-watch/) that aggregates NFL injury data from NFL insiders, team websites and NFL injury reports. According to the 2016 NFL Injury Report Policy, each team is responsible for reporting any “significant or noteworthy injury” in their practice reports, in their game status reports, and during in-game status reports to the public [3]. The injury report from the Concussion Watch Injury Reports includes the following information from the players with head injury: injury type, week of injury, average play in games before the injury, play time in the return game, games missed, description of the injury, and whether the player was immediately removed from the game.

The NFL regular season consists of 16 games, played among 32 teams. Since the NFL does not mandate non-playoff teams to share injury reports for the last week of the season (week 17), we excluded week 17 data from our analysis. Additionally, NFL teams do not necessarily share all the self-reported concussion injuries sustained during practice. Therefore, we did not include head trauma and concussion injuries reported from the practice sessions. We also did not include pre-season and playoff concussions in our analysis.

Our study utilized PBS FRONTLINE Concussion Watch to (FCW) to collect our data retrospectively from the four regular seasons (2012 to 2015) for weeks 1 to 16. All injuries described as “concussion” or “head injuries” by the PBS FCW were used in our analysis.

Meteorological mean game-day data for the city was collected retrospectively for each game-day using meteorological archives (www.wunderground.com). Our variables included temperature, humidity, barometric pressure, and dew point.

Concussion incidence and risk

We calculated concussion incidence and probability of a concussion game using the following definitions.

Concussion Incidence

Total # of Concussions/Total # Games for a specific environmental variable

Probability of a Concussion Game

Total # of Concussion Games/Total # of Games for a Specific environmental variable

We also calculated and recorded the mean game-day temperature, humidity, dew point, and barometric pressure between mean concussion and concussion-free games.

Temperature

The mean game-day temperature quartiles used for analysis were <=40^o^F, 41-55^o^F, 56-70^o^F, and >70^o^F. An additional analysis was performed using 20^o^F increments (1-20^o^F, 21-40^o^F, 41-60^o^F, 61-80^o^F, and 81-100^o^F).

Humidity

The mean game-day humidity quartiles used for analysis were <60%, 61-70%, 71-80%, and >80%.

Barometric Pressure

The mean game-day barometric pressure quartiles used for analysis were <30.00 inHg, 30.01-30.10 inHg, 30.11-30.20 inHg, and >30.20 inHg.

Dew Point

The mean-game day dew-point quartiles used for analysis were <30^o^F, 30-45^o^F, 46-60^o^F, and >60^o^F.

Statistical analysis

Besides descriptive statistics, we performed a correlation analysis for all the aforementioned environmental factors. A t-test and ANOVA were performed using GraphPad Prism 6 (GraphPad Software, La Jolla, CA, US) on all four environmental factors and their role in the incidence of concussion. Poisson regression and logistic regression were performed using RStudio (Boston, MA, US) on all four environmental factors to compute the predicted incidence and probability of concussion, respectively. All variables identified with an initial significance value of alpha level < 0.05 were considered significant.

## Results

Concussions

A total of 960 regular season games played during the 2012 to 2015 seasons from week one to week 16 were included in this study. In four seasons, there were 564 concussions in 960 games. There were 411 games with concussions (43%) and 549 games (57%) that were concussion-free.

Environmental factors 

The rate of concussion incidence and risk of concussion incidence were assessed in four different environmental variables: temperature, dew point, humidity, and barometric pressure (Table 1).

**Table 1:** Demographic Data for 2012-2015 NFL Seasons NFL: National Football League

Demographics Data of Included Team Games (N = 960)					
Variable	n	% out of 960 games	Mean Concussion Incidence	Confidence Interval	Standard Error
Mean Game Day Temperature Range in ^o^F (20^o^F Increments)					
< 20^o^F	11	1.15%	1.00	0.15 – 1.85	0.3814
21 to 40^o^F	134	13.96%	0.7313	0.58 – 0.88	0.07796
41 to 60^o^F	418	43.54%	0.6483	0.56 – 0.72	0.04102
61 to 80^o^F	362	37.71%	0.4751	0.40 - 0.54	0.0361
> 80^o^F	35	3.65%	0.3429	0.12 – 0.56	0.108
Mean-Game Day Temperature (Quartile Analysis)					
< 40^o^F	145	15.10%	0.7517	0.59 – 0.90	0.07739
40 to 55^o^F	279	29.06%	0.6918	0.59 – 0.79	0.05147
55 to 70^o^F	363	37.81%	0.5041	0.42 – 0.58	0.0379
> 70^o^F	173	18.02%	0.4566	0.35 – 0.56	0.05279
Mean-Game Day Humidity					
< 60%	273	28.44%	0.6190	0.52 – 0.71	0.04956
61 to 70%	259	26.98%	0.5444	0.44 – 0.64	0.04927
71 to 80%	253	26.35%	0.5968	0.50 – 0.69	0.04865
> 80%	175	18.23%	0.5886	0.47 – 0.71	0.06235
Mean-Game Day Barometric Pressure					
< 30 inHg	374	38.96%	0.6070	0.52 – 0.69	0.04176
30.01 to 30.10 inHg	211	21.98%	0.5308	0.42 – 0.63	0.05129
30.11 to 30.20 inHg	181	18.85%	0.4641	0.36 – 0.56	0.05343
> 30.20 inHg	194	20.21%	0.7268	0.60 – 0.85	0.06424
Mean-Game Day Dew Point					
< 30^o^F	203	21.15%	0.6650	0.55 – 0.77	0.05526
30 to 45^o^F	271	28.23%	0.6679	0.55 – 0.78	0.05705
45 to 60^o^F	343	35.73%	0.5685	0.48 – 0.64	0.04068
> 60^o^F	143	14.90%	0.3706	0.27 – 0.46	0.04829

Temperature

**Figure 1:**
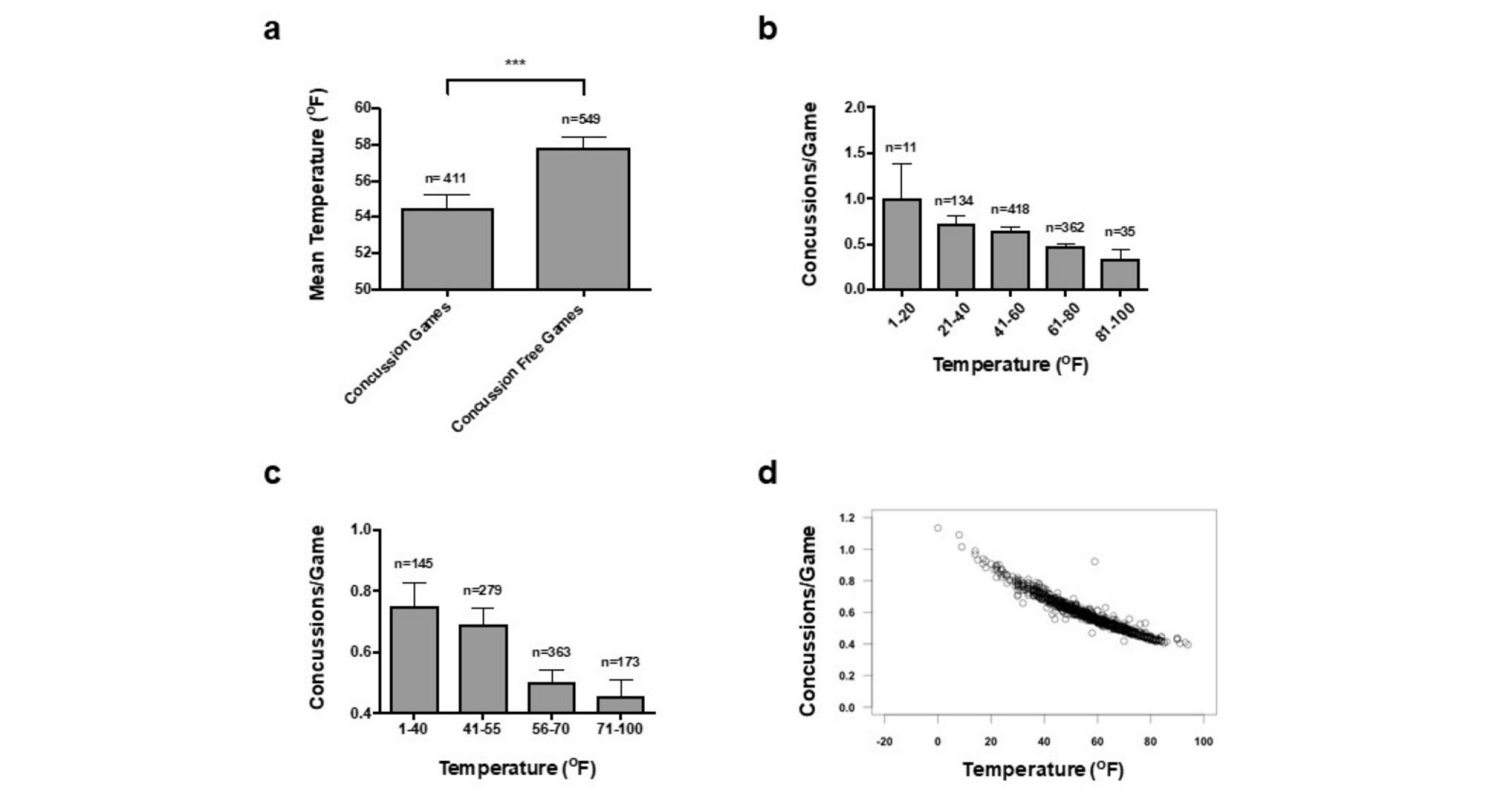
Concussion Incidence Increases as Game Temperature Decreases (a) Bar graph shows mean temperature (SEM) for concussion and concussion-free games (p <0.001, ***). (b-c) Bar graphs show mean concussion incidence for games played at different temperature increments. Graph b divides temperature into 20^o^F increments, and graph c divides temperature into 15^o^F and 30^o^F increments. (d) Poisson regression computes the predicted concussion incidence of any given game played at a certain temperature. The environmental factors of temperature, dew point, humidity, and barometric pressure were utilized in the model.

There was a significant difference in the mean game-day temperature between concussion and concussion-free games. In 411 concussion games, the mean game-day temperature was 54.48 ± 0.7421^o^F (SEM); meanwhile, the average temperature for 549 concussion-free games was 57.82 ± 0.6276^o^F (p = 0.0006; Figure 1a). One-way ANOVA showed that there was a significant decrease in concussion incidence with increasing temperature both when the temperature was divided in 20^o^F increments (p = 0.005) and when the temperature was divided within the quartile analysis (p = 0.002, Figures 1b-1c). In the quartile analysis, with the exception of comparisons 1-40^o^F versus 41-55^o^F and 56-70^o^F versus >70^o^F, Tukey’s multiple comparison test showed significant differences between all the remaining quartile analysis: 1-40^o^F and 55-70^o^F (p = 0.0084), 1-40^o^F and >70^o^F (p = 0.0055), 44-55^o^F and 56-70^o^F (p = 0.016), and 40-55^o^F and >70^o^F (p = 0.0121). Poisson regression, taking into account all four factors, was also performed on the dataset to compute the predicted concussion incidence of any given game played at a certain temperature (Figure 1d).

**Figure 2:**
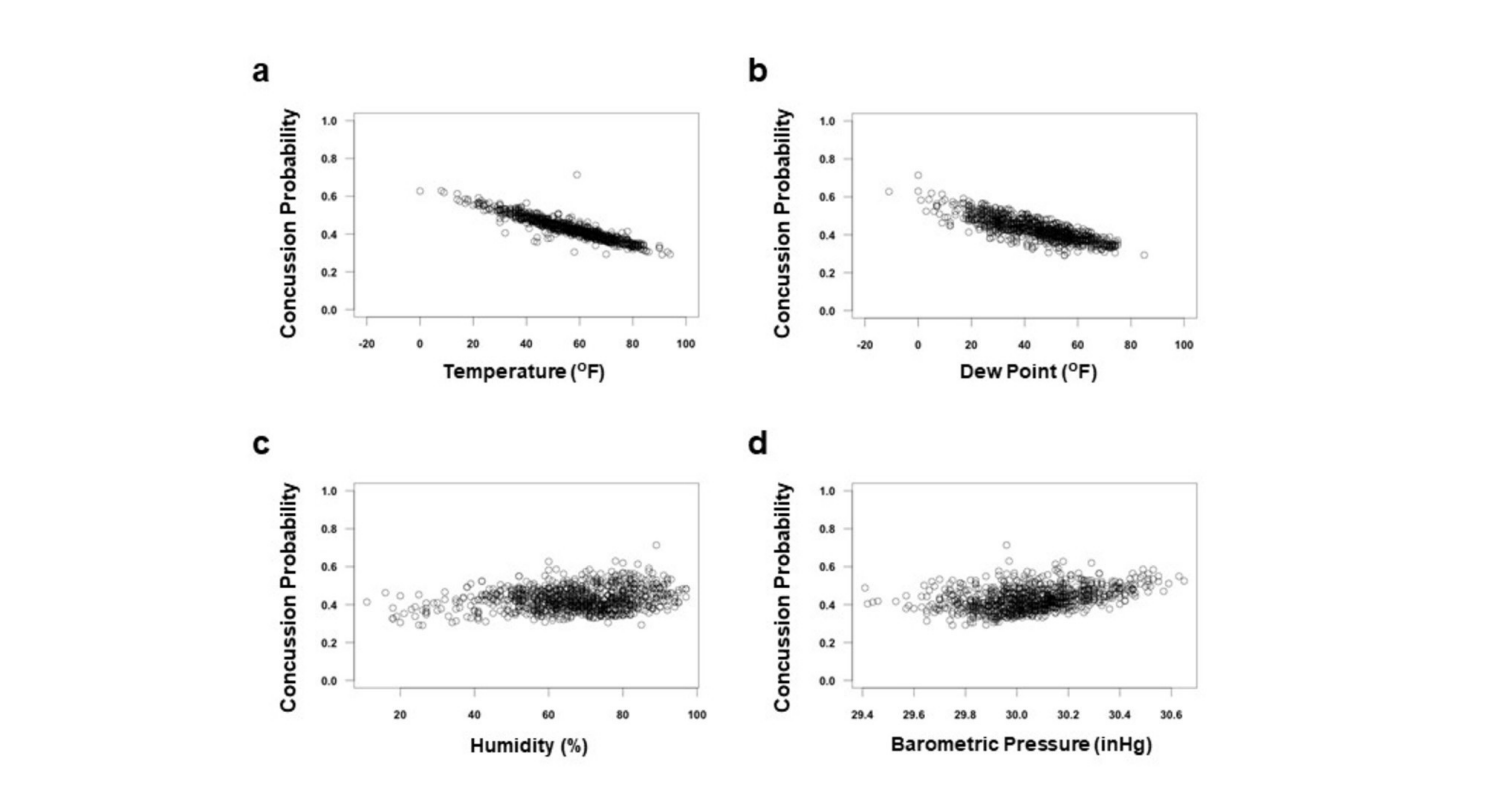
Lower Temperature and Dew Point Is Predictive of Concussion Incidence (a-d) Logistic regression, taking into account all four environmental factors, was performed on the total 960 games to compute the predicted probability that a concussion would occur during a game. Graphs a, b, c, and d show temperature, dew point, humidity, and barometric pressure, respectively.

We identified a higher incidence at temperatures below 55^o^F. When played below 55^o^F, the mean concussion/game was 0.7017 ± 0.04335^o^F, and when played above 55^o^F, the mean concussion per game was 0.5027 ± 0.03103^o^F (p = 0.0001). Logistic regression, taking into account all four factors, was performed on the dataset to compute the predicted probability that a concussion would occur during a game for any given temperature (Figure 2). 

Dew Point

**Figure 3:**
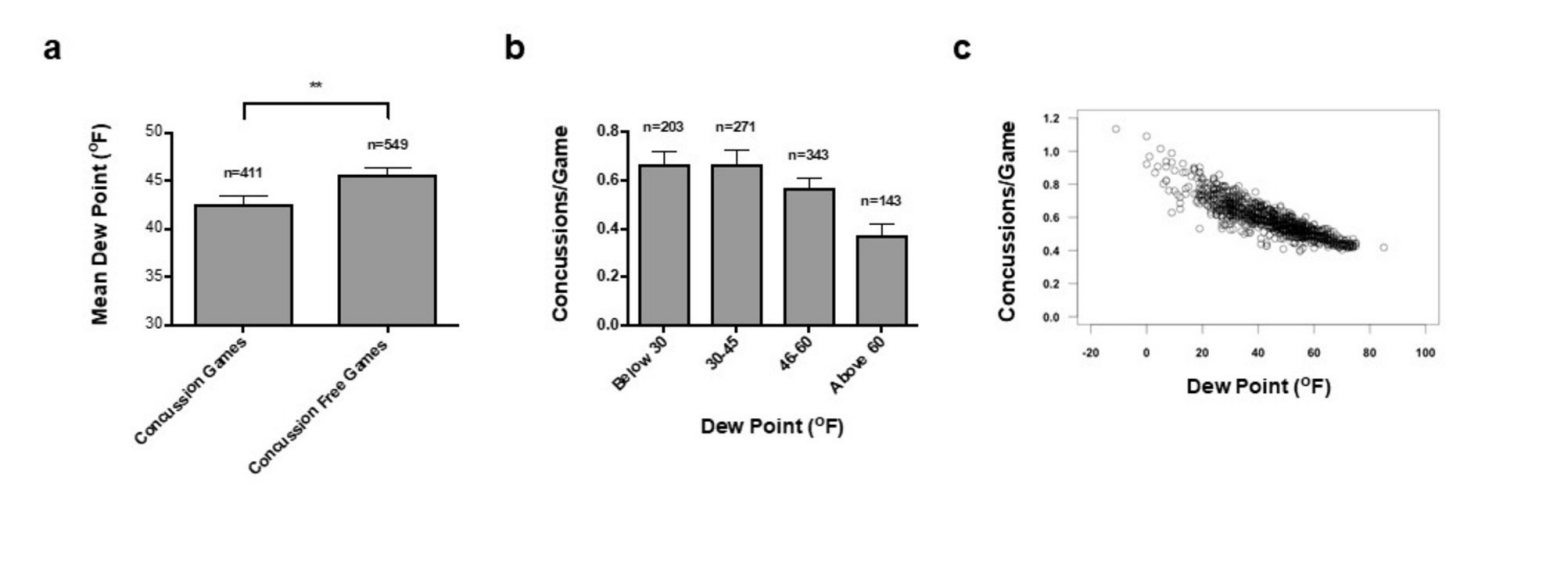
Concussion Incidence Increases as Dew Point Decreases (a) Bar graph shows mean dew point (SEM) for concussion and concussion-free games (p<0.01, **). (b) Bar graph shows mean concussion incidence for games played at different dew point increments. (c) Poisson regression computes the predicted concussion incidence of any given game played at a certain dew point. The environmental factors of temperature, dew point, humidity, and barometric pressure were utilized in the model.

The total 960 games were assessed with specific ranges of dew point temperature. There was a significant difference between the mean dew point temperature of concussion (42.64 ± 0.7591^o^F) and concussion-free games (45.64 ± 0.6495^o^F) (p = 0.0026; Figure 3a). Secondly, the quartile analysis of 960 games for dew point was performed to identify changes in the rate of concussion. One-way ANOVA testing showed a significant decrease in the incidence of concussion per game with increasing dew point above 60^o^F (p = 0.0015). Tukey’s multiple comparison showed significant difference between <30^o^F and >60^o^F groups (p = 0.0040) and between 30-45^o^F and >60^o^F (p = 0.0018; Figure 3b). Poisson regression, taking into account all four factors, was also performed on the dataset to compute the predicted concussion incidence of any given game played at a certain dew point (Figure 3c). Logistic regression, taking into account all four factors, was performed on the dataset to compute the predicted probability that a concussion would occur during a game for any given temperature (Figure 2). Our correlation analysis suggested a strong correlation between temperature and dew point (Rho = 0.86).

Humidity and Pressure

**Figure 4:**
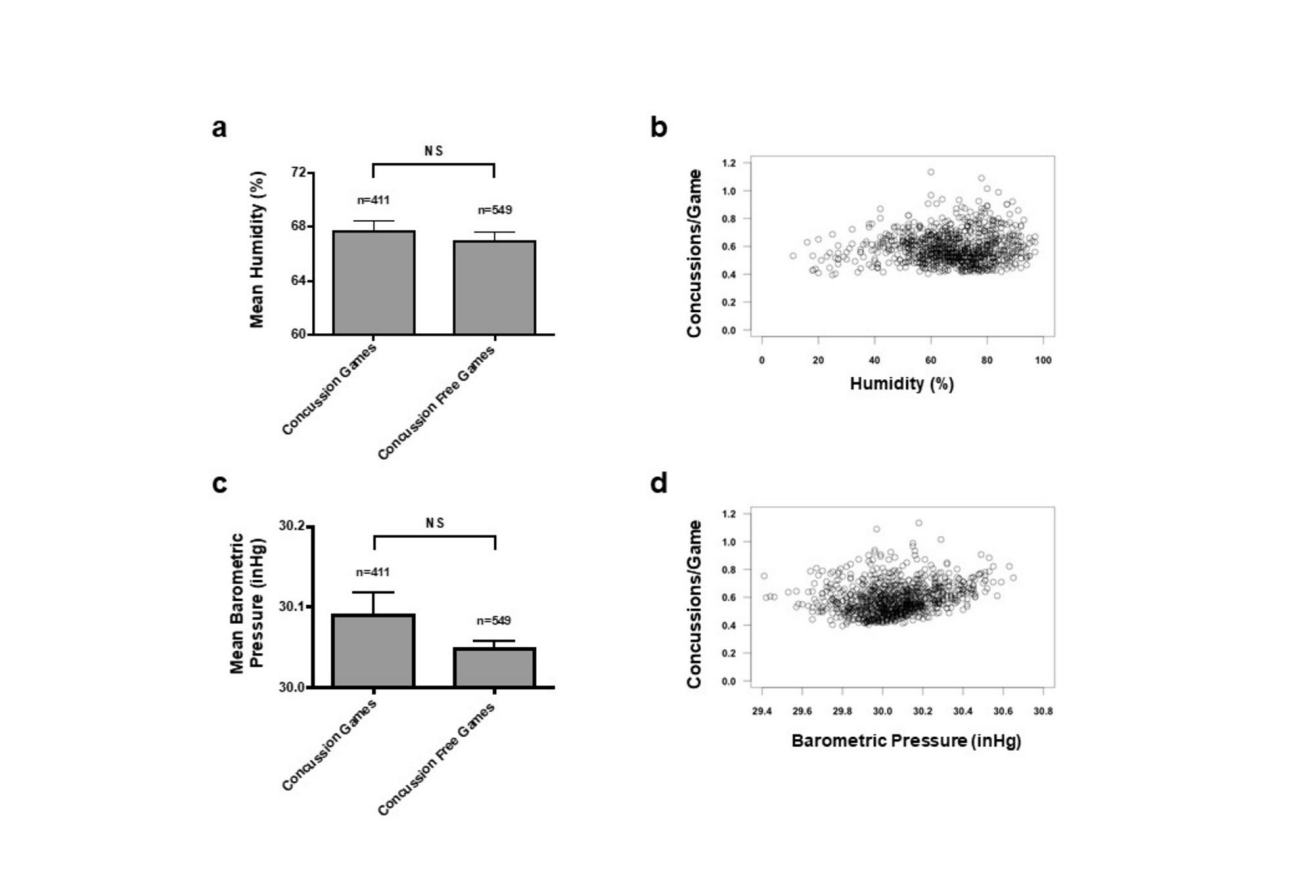
Humidity and Barometric Pressure Do Not Affect Concussion Incidence (a) Bar graph shows mean dew point (SEM) for concussion and concussion-free games (p>0.05, NS). (b) Poisson regression computes the predicted concussion incidence of any given game played at a certain humidity. The environmental factors of temperature, dew point, humidity, and barometric pressure were utilized in the model. (c) Bar graph shows mean barometric pressure (SEM) for concussion and concussion-free games (p>0.05, NS). (d) Poisson regression computes the predicted concussion incidence of any given game played at a certain barometric pressure. The environmental factors of temperature, dew point, humidity, and barometric pressure were utilized in the model.

The total 960 games were assessed with a specific range of humidity and barometric pressure. There was no significant difference for mean humidity and mean barometric pressure between concussion and concussion-free games (Figure 4). In 411 concussion games, the mean humidity was 67.70 ± 0.7016% and mean barometric pressure was 30.09 ± 0.026 inHg. In 549 concussion-free games, the mean humidity was 66.9 ± 0.64% and mean barometric pressure was 30.05 ± 0.0079 inHg. (Figures 4a-4c). Poisson regression and logistic regression, taking into account all four factors, were also performed on the dataset to compute the predicted concussion incidence and probability that a concussion would occur during a game, respectively, at a certain humidity or barometric pressure (Figures 2-4). There were no significant changes in the rate of concussion incidence for various ranges of humidity and barometric pressure (Figures 4b-4d).

Risk of concussion

Using Poisson regression, the total 960 games were used to predict the number of concussions during a game based on the four environmental factors (Figure 1, Figures 3-4). There were no significant predictors when all four environmental factors were considered together; this is likely due to the strong correlation between temperature and dew point, thus making it difficult to distinguish the true coefficient and increasing the standard error. Therefore, Poisson regressions were performed with either temperature or dew point and the other two factors. Excluding dew point, the model predicted the effect of an increase of temperature of 1^o^F is a decrease in concussions of –0.014427. Excluding temperature, the model predicted the effect of an increase of dew point of 1^o^F is a decrease in concussions of –0.016143 (Figures 1-3).

Similar results were found in the logistic regression of the 960 games to model the predicted probability that a concussion would occur during a game (Figure 2, Table 2). There were no significant predictors when all four environmental factors were considered together likely due to the strong correlation between temperature and dew point. Excluding dew point, the model predicted the effect of an increase of temperature of 1^o^F is a –0.012105 decrease in the probability of concussions. Excluding temperature, the model predicted the effect of an increase of dew point of 1^o^F is a –0.012806 decrease in the probability of concussions.

**Table 2:** Logistic and Poisson Regression Outputs for 2012-2015 National Football League Seasons

Logistic Regression				Poisson Regression			
	Estimate (β)	Standard Error	P		Estimate (β)	Standard Error	P
Intercept	-3.699928	11.263321	0.743	Intercept	2.790439	7.013908	0.691
Temperature	0.004887	0.017456	0.779	Temperature	-0.004127	0.010328	0.689
Humidity	0.012402	0.009654	0.199	Humidity	0.003784	0.005705	0.507
Pressure	0.0197517	0.369371	0.771	Pressure	-0.098979	0.22995	0.667
Dew	-0.021079	0.018338	0.25	Dew	-0.008686	0.010785	0.421
	Estimate (β)	Standard Error	P		Estimate (β)	Standard Error	P
Intercept	-3.122559	11.241082	0.78118	Intercept	3.0350837	7.0062927	0.665
Temperature	-0.014427	0.004738	0.00233	Temperature	-0.012105	0.0029258	3.51E-05
Humidity	0.002652	0.004558	0.56069	Humidity	-0.0001565	0.0029554	0.958
Pressure	0.115266	0.368978	0.75474	Pressure	-0.0961313	0.2299181	0.676
	Estimate (β)	Standard Error	P		Estimate (β)	Standard Error	P
Intercept	-3.235476	11.138963	0.77146	Intercept	2.355913	6.936975	0.734
Humidity	0.010072	0.004868	0.03853	Humidity	0.005698	0.003065	0.063
Pressure	0.099163	0.36086	0.78762	Pressure	-0.090463	0.229153	0.693
Dew	-0.016143	0.004978	1.18E-03	Dew	-0.012806	0.003047	2.64E-05

## Discussion

This study identified the environmental risk factors associated with head injuries in the NFL over the course of four seasons from 2012 to 2015. First, we provided support for a previously debated finding and identified a significant trend of reduction in concussion incidence with increasing temperature. Second, we identified additional risk factors and their association with head injuries and concussion. We observed an increasing incidence of concussion at colder dew point temperatures and determined no significant effect of barometric pressure and humidity on the rate of concussion incidence. Third, we identified and calculated the risk of concussion games at various environmental ranges, providing a tool for researchers, parents, coaches, and professional athletes to understand the possibility of a concussion game during different environmental conditions. These findings, when understood in context, can be used to educate NFL teams, organizations, sports leagues, and medical communities about the impact of various environmental conditions on concussion risk and incidence.

Our first major goal was to identify and validate the relationship between increasing game day temperature and decreasing concussion rate. A thorough literature review revealed conflicting data regarding the role of temperature in concussion incidence. A study by Lawrence et al. in 2016 identified a greater risk of concussion for games played at a mean game-day temperature of ≤49.5°F and a trend showing increasing risk with decreasing temperature based on two NFL seasons [7]. Other studies showed no meaningful correlation between temperature and increased prevalence of concussions. Orchard et al. in 2013 showed no significant association between the prevalence of head injuries and temperature in soccer games played in three professional soccer leagues [1]. A study conducted by Karnatovskaia et al. in 2014 reported that hypothermia triggered neuroprotective mechanisms that affected head trauma and decreased inflammation to positively preserve the brain blood barrier [12]. Hence, different physiological theories have been put forth to explain these conflicting conclusions. Our study examined the difference in mean temperature between concussion and concussion-free games and evaluated the concussion incidence rate against temperature. We observed that the average temperature was lower in concussion games, and there was a general decreasing trend of concussion incidence with increasing temperature validated over four NFL seasons. For temperature, in particular, we identified a statistically significant reduction in concussion incidence from a colder temperature to a higher temperature regardless of how the temperature ranges were divided (20^o^F increments versus quartile analysis). These two findings suggest that the warmer temperatures may have neuroprotective effects on athletes.

Another aim for this study was to explore other environmental variables and expand this novel knowledge to include the impact of other extrinsic factors on head injuries. In the past, previous studies have only examined the role of altitude and distance traveled. After a thorough literature review, we identified no studies that examined the role of humidity, barometric pressure, and dew point on concussion incidence. Our study further examined these additional environmental variables and found a significant decrease in concussion incidence at increased dew point temperatures. We observed no significant difference in concussion incidence in various ranges of humidity and barometric pressure. Future studies should continue to assess how various other environmental and on-field factors contribute to the prevalence of concussions.

Several speculations might explain the increase in the incidence of concussions in the NFL with different environmental factors. The playing surface plays a pivotal role in the incidence of concussion. In a recent report in 2015, the Concussion Legacy Foundation found an association between head injuries and poorly maintained fields [13]. According to the report, 15.5% of concussions in high school sports is the result of hitting the head on the playing surface [14]. Thus, more attention ought to be given to how these surfaces, both grass and synthetic turf, are affected by environmental factors. For example, colder temperatures might lead to less compressible surfaces, resulting in harder impacts upon contact. It is possible that variations in or a lack of maintenance of natural or synthetic playing surfaces could adversely affect their mechanical properties and could contribute to the higher incidence of concussions in games being played at lower temperatures. Future studies should assess the difference in the effect of cold temperatures on fields of natural grass in comparison to synthetic turf.

Furthermore, another theory might describe the effects of temperature on cerebral inflammatory processes occurring in the brain during injury and trauma. Exposure to colder temperatures has been thought to depress immune function by reducing the expression and activation of astrocytes, microglial activation, and inflammatory cytokines [15]. While there is limited data examining the effect of cold exposure during times of physical activity, it is speculated that colder temperatures predispose the brain to diminished local immune response.

Our current study has several limitations. Data were gathered and analyzed from team-reported injury data. In the case of any self-reported data, there could be a bias in self-reporting, especially since teams hope to have their players return to play as quickly as possible. Next, the lack of precise past medical history and dates of injury for athletes also prevent us from understanding the impact of previous concussions on the player. In addition, limited information about practice concussions was reported, thereby restricting our data to game-day concussions only. Lastly, there are eight NFL teams that play their games under covered and retractable domes. An analysis revealed no significant difference between overall concussion incidence and concussion incidence beneath the dome. However, insufficient data prohibited a thorough understanding of the impact of domed stadiums on field game-day temperature.

## Conclusions

While there is great interest in concussion prevention and treatment, there is limited research on the exact environmental and extrinsic factors that affect its incidence. Our work expands upon current research by further highlighting that weather variables, including temperature and dew point, may play an important role in the incidence of brain injury. These data provide insight into the expanding knowledge of concussion risk and create a more complete understanding of environmental impact. By providing substantiated data, we hope to contribute to a useful and accessible tool by which leagues can assess individualized risk and thus take the necessary safety precautions to protect their players from head trauma.
